# An Expeditious Total Synthesis of 5′-Deoxy-toyocamycin and 5′-Deoxysangivamycin

**DOI:** 10.3390/molecules24040737

**Published:** 2019-02-19

**Authors:** Xiangyou Dong, Jie Tang, Chen Hu, Jiang Bai, Haixin Ding, Qiang Xiao

**Affiliations:** Key Laboratory of Organic Chemistry in Jiangxi Province, Institute of Organic Chemistry, Jiangxi Science & Technology Normal University, Nanchang 330013, China; dongxiangyou93@yeah.net (X.D.); tangjie93@yeah.net (J.T.); huchen92@yeah.net (C.H.); mtbaijiang@yeah.net (J.B.)

**Keywords:** nucleoside, 7-deazapurine, pyrrolo[2,3-*d*]pyrimidine, total synthesis

## Abstract

In present paper, an expeditious total synthesis of naturally occurring 5′-deoxytoyocamycin and 5′-deoxysangivamycin was accomplished. Because of the introduction of a benzoyl group at *N*-6 of 4-amino-5-cyano-6-bromo-pyrrolo[2,3-*d*]pyrimidine, a Vorbrüggen glycosylation with 1,2,3-tri-*O*-acetyl-5-deoxy-β-D-ribofuranose afforded a completely regioselective *N*-9 glycosylation product, which is unambiguously confirmed by X-ray diffraction analysis. All of the involved intermediates were well characterized by various spectra.

## 1. Introduction

The synthesis and biological evaluation of nucleoside/tide analogs as agents to treat cancers, viruses, and parasites, as well as fungal and bacterial infections, spans the past half century [1,2,3]. The first steps in this field can be traced back to the emergence of cytarabine to treat for acute myeloid leukemia and edoxudine for herpes simplex virus, which were both approved by the FDA in 1969. This strategy has become very successful and there are now more than 30 nucleoside/tide analogues approved for clinic use, with many more ongoing in preclinical and clinical trials [4,5,6].

**Figure 1:**
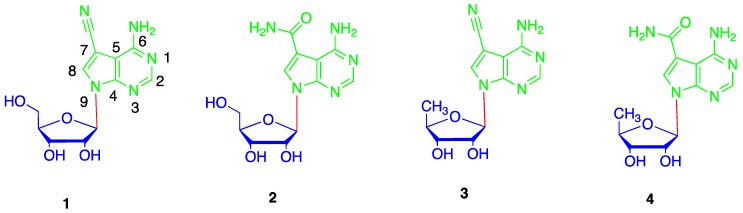
Examples of naturally occurring 7-deazapurine nucleosides.

As important analogues of biogenic purine nucleosides, 7-deazapurine nucleosides have shown diverse biological activities [7,8,9,10]. Replacement of the purine’s *N*-7 atom with a carbon atom results a more electron rich pyrrole ring more prone to cation–π or π–π interactions, which subsequently leads to increased nucleobase-pairing in DNA or RNA and possibly better binding to proteins [11,12]. Many 7-deazapurine nucleosides were isolated as naturally occurring products, such as toyocamycin (**1**) and sangivamycin (**2**) from *Streptomyces* cultures (Figure 1). They showed potent cytotoxicity against various cancer cell lines with different modes of action. Moreover, toyocamycin (**1**) was identified recently as a potent inhibitor of induced XBP1 mRNA cleavage, which is a key component of the endoplasmic reticulum stress response [13]. On the other hand, the cytotoxic effect of sangivamycin (**2**) is mainly due to selective inhibition of protein kinase C through inhibition of *Erk* and *Akt* signaling pathway [14,15].

In the course of our continued research interest in the synthesis of 7-deazapurine nucleosides and their biological activities, 5′-deoxytoyocamycin (**3**) and 5′-deoxysangivamycin (**4**) (Figure 1) were needed as a control to compare their biological action mechanism with toyocamycin (**1**) and sangivamycin (**2**), respectively. In the published literature, 5′-deoxytoyocamycin (**3**) and 5′-deoxysangivamycin (**4**) were originally synthesized in moderate yields from toyocamycin and sangivamycin by radical reduction (AIBN and tributyltin hydride in dry THF) of the corresponding 5′-chloro-5′-deoxynucleosides [16,17]. This approach was inefficient, cumbersome and low yielding and the the starting materials very expensive. Later on, 5′-deoxytoyocamycin (**3**) was isolated from the fermentation broth of a *Streptomyces* sp. from a soil sample collected from Kepong, Malaysia. Although a manufacturing process for 5’-deoxytoyocamycin using the *Streptomyces* sp. was patented soon after its discovery, it was not practical to perform it in our laboratory. Thus, we turned our endeavor to total syntheses of 5′-deoxytoyocamycin (**3**) and 5′-deoxysangivamycin (**4**), which could provide enough material for further biological studies.

**Figure 2:**
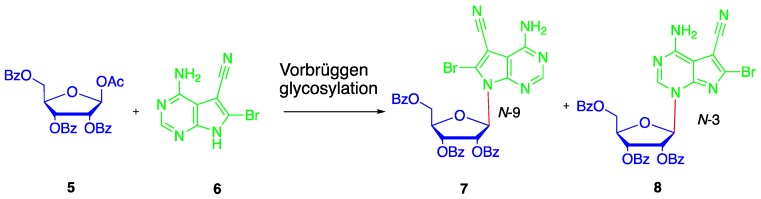
Synthesis of 7-deazapurine nucleosides by Vorbrüggen glycosylation.

While various glycosylation approaches have been developed for the preparation of ribonucleosides, the most widely employed method is the silyl-Hilbert-Johnson reaction catalyzed by equimolar or excess amounts of Lewis acid catalysts (Vorbrüggen glycosylation), which has now been developed for more than fifty years [18]. However, the Vorbrüggen glycosylation when performed with 7-deazapurines was found to be much less efficient. Until now, Vorbrüggen glycosylation could only be used to prepare the corresponding ribonucleosides of 7-deazapurine nucleobases with an electron withdrawing substitute at C-7 [19]. Specifically, total synthesis of toyocamycin (**1**) by Vorbrüggen glycosylation of ribose **5** and nucleobase **6** was firstly reported by Bobek [20], and further improved by Townsend [21], but during our many repeats of this approach [22,23,24,25], it was found that the *N*-9 glycosylation product **7** and *N*-3 glycosylation product **8** were inevitably formed simultaneously (Figure 2). This phenomenon was also reported by several other groups [26,27,28]. The selectivity and equilibrium between *N*-9 glycosylation product **7** and *N*-3 glycosylation product **8** were subsequently studied in detail [27]. In order to resolve this problem, an improved synthetic approach is reported in the present paper, that was used successfully in the total synthesis of 5′-deoxytoyocamycin (**3**) and 5′-deoxysangivamycin (**4**).

## 2. Results and Discussion

According to the reaction mechanism of the Vorbrüggen glycosylation, we reasoned that *N*-3 product formation may result from the more electron rich pyrimidine in nucleobase **6**. Thus, the formation of *N*-3 products, such as nucleoside **8**, could be avoided by reducing the pyrimidine ring’s electron density. On the other hand, the solubility of nucleobase **6** is very low and its purification is really difficult. We speculated that nucleobase **12** with a benzoyl group at *N*-6 could fulfill our requirements. Although, the nucleobase **6** with an acetyl at *N*-6 was already reported, its use did not solve the above selectivity and solubility problems [20,29,30,31].

**Scheme 1:**
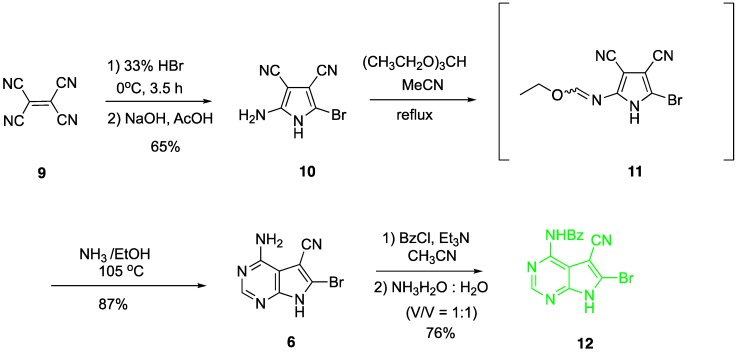
Synthesis of *N*^4^-benzoyl-5-cyano-6-bromo-7*H*-pyrrolo[2,3-*d*]pyrimidine.

Preparation of nucleobase **6** from tetracyanoethylene **9** was reported in several references [32,33,34,35]. In order to synthesize it on a large scale, the corresponding synthetic procedure was improved in our following experiments (Scheme 1). Firstly, pyrrole **10** were synthesized from tetracyanoethylene **9** according to a modification of the protocol developed by Townsend [36]. It should be mentioned that the intermediate formed after addition of 33% HBr is very hydroscopic and not stable in air. After transformation to the free pyrrole **10**, it could be stored on the bench for a long time. Then, pyrrole **10** was refluxed with ethyl orthoformate in CH_3_CN to give intermediate **11**. After removing the volatile byproducts and without further purification, ring annulation to give nucleobase **6** in 87% yield was accomplished in freshly prepared saturated ethanolic ammonia at 105 °C in a closed stainless-steel container for 6 h. 

**Figure 3:**
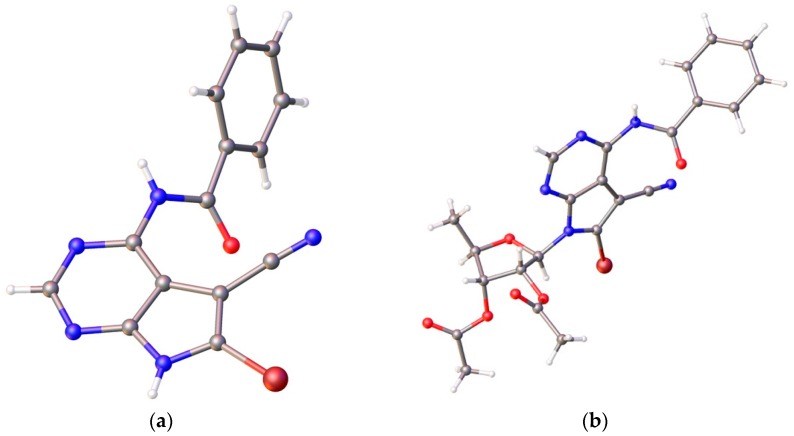
X-Ray structure of nucleobase **12** (**a**) and nucleoside **14** (**b**).

The subsequent monobenzoylation at *N*-6 to provide nucleobase **12** proved to be troublesome. Using pyridine as the base and solvent, at least four products are formed. Other attempts to directly synthesize the monobenzoylated product **12** also failed. After optimization, complete benzoylation of nucleobase **6** was conducted firstly to give the corresponding *N*6,*N*6,*N*9-tribenzoyl derivative of **6** using a large excess of benzoyl chloride (5 eq.) and Et_3_N (7.5 eq) in CH_3_CN. Then the tribenzoyl intermediate, without isolation, was stirred directly with concentrated ammonia and H_2_O (*V*/*V* = 1:1) to afford the desired nucleobase **12** in 76% yield. This modified protocol for the synthesis of nucleobase **12** from **9** was repeated several times at 50 g scale without requiring chromatographic purification. Furthermore, a single crystal of nucleobase **12** suitable for X-ray crystallographic analysis was obtained and the X-ray structure shown in Figure 3a was determined [37].

**Scheme 2:**
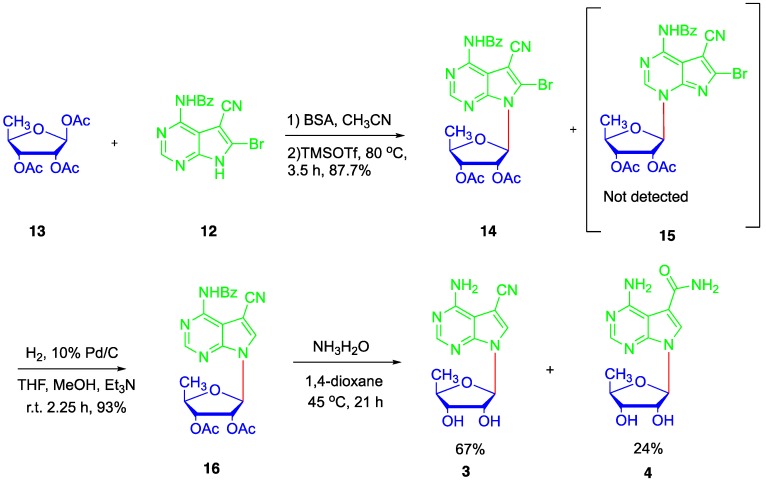
Total synthesis of 5′-deoxytoyocamycin and 5′-deoxysangivamycin.

Next, the Vorbrüggen glycosylation between ribose **13** and nucleobase **12** was conducted (Scheme 2). After silylation of nucleobase **12** with *N*,*O*-Bis(trimethylsilyl)acetamide (BSA) in dry MeCN, 1,2,3-tri-*O*-acetyl-5-deoxy-β-D-ribofuranose (**13**) was added, followed by TMSOTf. The resulting mixture was heated at 80 °C for 3.5 h. After purification, 7-deazapurine nucleoside **14** was obtained in 87.7% yield. To our delight, the *N*-3 glycosylation product **15** was not detected. Thus, our introduction of a benzoyl group at *N*-6 successfully solved the regioselectivity problem of the glycosylation. At the same time, a single crystal of nucleoside **14** suitable for X-ray diffraction analysis was also obtained and its structure is unambiguously confirmed, which showed an *anti*-glycosylic bond conformation and an envelope C2′-*end*o (^2^*E*) sugar conformation (S-type) (Figure 3b) [36]. It is worth mentioning that X-ray structures of toyocamycin and its analogues have seldom been reported [38].

Subsequently, the bromide at C-8 was hydrogenated in a solvent mixture of methanol and THF using 10% Pd/C as catalyst to give nucleoside **16** in 93% yield. Deprotection of the remaining four benzoyl groups proved troublesome. When heated with saturated ammonium in methanol, 5′-deoxytoyocamycin (**3**) and 5′-deoxysangivamycin (**4**) were obtained at the same time along with other side products.

A variety of other conditions were tried with no improvement. Looking back the X-ray structures of nucleobase **12** and nucleoside **14**, it was obvious that the carbonyl of the *N*-6 benzoyl was close and *syn* to the C-7 nitrile group (Figure 3) [37]. Therefore, the nitrile group of nucleoside **16** could be activated by the electron-withdrawing benzoyl group, which makes the nitrile group susceptible to attack by ammonia. Luckily, 5′-deoxytoyocamycin (**3**) and 5′-deoxysangivamycin (**4**) were both targets, and they could be conveniently separated by chromatography. In the end, 5′-deoxytoyocamycin (**3**) and 5′-deoxysangivamycin (**4**) were obtained in 67% and 24% yield, respectively, after removing all the benzoyl protecting groups with ammonia in dioxane. All the spectra of 5′-deoxytoyocamycin (**3**) were in accordance with those of the naturally occurring product. Because different solvents were used in the original NMR determination of naturally occurring 5′-deoxytoyocamycin (**3**) [39], the corresponding NMR data obtained in various solvents are summarized in this work (Table S1 in the Supplementary Information).

## 3. Conclusions

In conclusion, we have accomplished an improved total synthesis of 5′-deoxytoyocamycin (**3**) and 5′-deoxysangivamycin (**4**) using a Vorbrüggen glycosylation between 1,2,3-tri-*O*-acetyl-5-deoxy-β-D-ribofuranose (**13**) and *N*^4^-benzoyl-5-cyano-6-bromo-7*H*-pyrrolo[2,3-*d*]pyrimidine (**12**) as the key step. The structure of 7-deazapurine nucleoside **14** was unambiguously confirmed by X-ray diffraction analysis, which showed an *anti* glycosylic bond conformation and an envelope C2′-*end*o (^2^*E*) sugar conformation. It was demonstrated that the regioselectivity during the Vorbrüggen glycosylation completely favored the *N*-9 glycosylation product after introducing a benzoyl group at the *N*-6 position of 4-amino-5-cyano-6-bromo-7*H*-pyrrolo[2,3-*d*]pyrimidine. Thus, this newly developed nucleobase **12** could be used to synthesize other toyocamycin analogues while avoiding the formation of *N*-3 glycosylation side products. 

## 4. Materials and Methods 

### 4.1. Materials and Instruments

Unless otherwise specified, all the reagents were acquired from commercial sources and used directly. NMR spectra (400 MHz/100 MHz) were recorded on an Advance DPX spectrometer (Bruker, Billerica, MA, USA) at room temperature with DMSO-*d*_6_ as solvent. Tetramethylsilane (TMS) was used as an internal reference. Specific rotations were acquired on an Autopol IV polarimeter (Rudolph, Hackettstown, NJ, USA). High resolution mass spectrometry (HRMS) data were measured with an AB Sciex TOF 4600 instrument (Billerica, MA, USA). Melting points were measured on an X-4 digital melting point apparatus (Beijing Taike Corparation, Beijing, China). X-ray diffraction analysis was performed on a Bruker Smart Apex II system (Bruker, Billerica, MA, USA). 

### 4.2. Synthesis of 2-amino-5-bromo-3,4-dicyano-1H-pyrrole *(**10**)*

Tetracyanoethylene (**9**, 35.6 g, 277.93 mmol) was dissolved in fresh dried acetone (200 mL) and ethyl acetate (420 mL). Under an Ar atmosphere, 33% HBr in acetic acid (200 mL) was added slowly while keeping the internal reaction temperature below 2 °C. After addition, the reaction mixture was stirred for another 1.5 h. The formed yellow solid was filtered and washed with cold ethyl acetate (100 mL). Subsequently, the crude product was suspended in ice cold water (100 mL) and 50% NaOH in H_2_O (60 mL) was added slowly until a homogenous solution was obtained. After filtration through Celite, the filtrate pH was adjusted carefully to 5 using acetic acid. The thus obtained solid was filtered and washed thoroughly with water until neutral. After drying under vacuum with P_2_O_5_, a grey solid of **10** was obtained (38.12 g, 65%). R*_f_* = 0.6 (DCM/MeOH, *V/V* = 10:1); mp: 224–226 °C; ^1^H-NMR δ: 12.25 (s, 1H), 6.45 (s, 2H); ^13^C-NMR δ: 149.8, 115.1, 114.4, 103.0, 93.5, 71.4; HRMS: calcd. for C_6_H_2_BrN_4_ [M − H]^−^ 208.9468; found: 208.9446.

### 4.3. Synthesis of 4-amino-5-cyano-6-bromo-7H-pyrrolo[2,3-d]pyrimidine *(**6**)*

2-Amino-5-bromo-3,4-dicyano-1*H*-pyrrole (**10**, 65.5 g, 310.40 mmol) was suspended in fresh dried CH_3_CN (1.5 L) and ethyl orthoformate (77.44 mL, 465.59 mmol). The suspension was heated to reflux until TLC showed the reaction was finished (about 3 h). The solvent was removed under vacuum to give a grey solid (82 g). Subsequently, the obtained solid was suspended in freshly prepared saturated ammonia in ethanol (1150 mL) in a closed stainless-steel container and heated at 105 °C for 6 h. Once cooled to room temperature, the reaction mixture was poured to a 5 L beaker with activated charcoal (22 g). After stirring for 30 min, the mixture was filtered through Celite and washed thoroughly with concentrated ammonia in ethanol (*V/V* = 1/5, 150 mL × 3). The filtration was adjusted to pH = 5 with acetic acid (about 160 mL). The obtained solid was filtered and washed with H_2_O. After dried under vacuum with P_2_O_5_, **6** was obtained as greenish solid (64.28 g, 87%). R*_f_* = 0.6 (DCM/MeOH, V/V = 10:1); mp: 279–281 °C; ^1^H-NMR δ: 8.21 (s, 1H, H-2), 7.20 (s, 2H, NH_2_); ^13^C-NMR δ: 156.0(C-4), 150.1(C-7a), 149.6 (C-2), 124.9 (C-6), 115.9 (CN), 103.7 (C-4a), 84.4(C-5); HRMS: calcd. for C_7_H_3_BrN_5_ [M−H]^−^ 235.9577; found: 235.9585.

### 4.4. Synthesis of N^4^-benzoyl-5-cyano-6-bromo-7H-pyrrolo[2,3-d]pyrimidine *(**12**)*

Deazapurine base **6** (50 g, 210.05 mmol, 1 eq.) was suspended in fresh dried CH_3_CN (450 mL) and Et_3_N (220 mL, 1.58 mol, 7.5 eq.). After the above suspension was stirred for 1 h at room temperature, benzoyl chloride (122 mL, 1.05 mol, 5 eq.) was added and the reaction mixture was stirred overnight. After TLC showed the 7-deaazapurine base had vanished, the reaction pH was adjusted by acetic acid to 6 and he reaction mixture was filtered. The filtrate was extracted with DCM (300 mL × 3). The combined extracts were washed with H_2_O, saturated NaCl, and dried with anhydrous MgSO_4_. After filtration, the solvent was evaporated under vacuum. The obtained crude product was stirred with concentrated ammonium and H_2_O (1 L, *V*/*V* = 1:1) for 5 h to give light yellow protected deazapurine **12** (54.63 g, yield: 76%). R*_f_* = 0.3 (CH_2_Cl_2_/MeOH, *V/V* = 10:1); mp 235–237 °C; ^1^H-NMR δ: 14.23 (s, 1H, NH), 11.36 (s, 1H, NH), 8.75 (s, 1H, H-2), 8.06 (d, *J* = 6.3 Hz, 2H), 7.65 (t, *J* = 7.2 Hz, 1H), 7.56 (t, *J* = 7.5 Hz, 2H); ^13^C-NMR δ: 167.4 (C=O), 153.3 (C-4), 152.7 (C-2), 150.6 (C-7a), 133.5, 132.9, 128.9, 128.8, 124.1 (C-6), 114.8 (CN), 111.9 (C-4a), 88.8 (C-5); HRMS: calcd. for C_14_H_7_BrN_5_O [M − H]^−^ 339.9839; found: 339.9852.

### 4.5. Synthesis of N^4^-benzoyl-5-cyano-6-bromo-7-(2′,3′-bi-O-acetyl-5′-deoxy-β-D-ribofuranosyl)-7H-pyrrolo[2,3-d]pyrimidine *(**14**)*

*N*,*O*-Bis(trimethylsilyl)acetamide (BSA, 14.27 g, 70.14 mmol) was added to a suspension of *N*^4^-benzoyl-5-cyano-6-bromo-7*H*-pyrrolo[2,3-d]pyrimidine (**12**, 6.00 g, 17.53 mmol) in dry MeCN (60 mL) under an argon atmosphere. The reaction mixture was stirred at room temperature for 60 min. A solution of 1,2,3-tri-*O*-acetyl-5-deoxy-β-D-ribofuranose **13** (5.48 g, 21.04 mmol) in dry MeCN (50 mL) along with TMSOTf (15.59 g, 70.14 mmol) were added to the above reaction mixture under ice-bath condition. The solution was stirred for 30 min at room temperature before heating to 80 °C for 3.5 h. Then the mixture was cooled and poured into sat. NaHCO_3_ solution (150 mL). It was extracted with CH_2_Cl_2_ (150 mL × 3). The combined organic layer was washed with sat. NaHCO_3_ (100 mL × 2), brine (100 mL × 2), and dried over anhydrous MgSO_4_. After filtration, the filtrate was evaporated to dryness under reduced pressure. The residue was purified by silica gel column chromatography (CH_2_Cl_2_ /EtOAc 20:1, *v*:*v*) to afford **14** as yellow solid (8.34 g, 87.7%). R*_f_* = 0.4 (CH_2_Cl_2_ /EtOAc, *V/V* = 20:1); m.p. 230–232 °C; $\alpha_{D}^{\mathrm{25}}$ −22.40 (c = 0.098, CH_2_Cl_2_); ^1^H-NMR δ 11.58 (s, 1H, NH), 8.89 (s, 1H, H-2), 8.06 (d, *J* = 7.5 Hz, 2H), 7.67 (t, *J* = 7.3 Hz, 1H), 7.57 (t, *J* = 7.6 Hz, 2H), 6.23 (d, *J* = 4.0 Hz, 1H, H-1′), 6.18–6.14 (m, 1H, H-2′), 5.50 (t, *J* = 6.7 Hz, 1H, H-3′), 4.33 – 4.25 (m, 1H, H-4′), 2.13 (s, 3H, OAc), 2.08 (s, 3H, OAc), 1.39 (d, *J* = 6.2 Hz, 3H, H-5’); ^13^C-NMR δ 170.1 (C=O, OAc), 170.0 (C=O, OAc), 167.5 (C=O, Bz), 153.1 (C-2), 152.0 (C-4), 151.4 (C-7a), 133.1, 129.0, 128.9, 126.6 (C-6), 114.2 (CN), 111.8 (C-4a), 91.4 (C-5), 89.1 (C-1′), 77.8 (C-4′), 74.0 (C-3′), 72.5 (C-2′), 20.8(OAc), 20.7 (OAc), 18.2 (C-5′); HRMS: calcd for C_23_H_21_BrN_5_O_6_ [M+H]^+^ 542.0670; found 542.0679.

### 4.6. Synthesis of N^4^-benzoyl-5-cyano-7-(2′,3′-di-O-acetyl-5′-deoxy-β-D-ribofuranosyl)-7H-pyrrolo[2,3-d]pyrimidine *(**16**)*

10% Pd/C (0.75 g, 0.70 mmol) and Et_3_N (0.94 g, 9.22 mmol) were added to a solution of nucleoside **14** (2.50 g, 4.61 mmol) in the mixture of THF (65 mL) and MeOH (35 mL). After stirring at room temperature for 2.25 h under H_2_ atmosphere (1 atm), the reaction mixture was filtered and concentrated to dryness under reduced pressure. The residue was purified by silica gel column chromatography (CH_2_Cl_2_/EtOAc = 20:1, *v*:*v*) to afford **16** as light yellow solid (1.99 g, 93%). R*_f_* = 0.3 (CH_2_Cl_2_ /EtOAc, *V/V* = 20:1); m.p. 92–94 °C; $\alpha_{D}^{\mathrm{25}}$ −45.80 (c = 0.109, CH_2_Cl_2_); ^1^H-NMR δ 11.50 (s, 1H, NH), 8.88 (s, 2H, H-2, H-6), 8.07 (d, *J* = 7.5 Hz, 2H), 7.66 (t, *J* = 7.2 Hz, 1H), 7.57 (t, *J* = 7.4 Hz, 2H), 6.40 (d, *J* = 5.4 Hz, 1H, H-1′), 5.90 (t, *J* = 5.6 Hz, 1H, H-2′), 5.32 (t, *J* = 5.2 Hz, 1H, H-3′), 4.32–4.26 (m, 1H, H-4′), 2.13 (s, 3H, OAc), 2.04 (s, 3H, OAc), 1.44 (d, *J* = 6.3 Hz, 3H, H-5′); ^13^C-NMR δ 170.0 (C=O, OAc), 169.8 (C=O, OAc), 167.5 (C=O, Bz), 153.2 (C-2), 152.5 (C-4), 152.1(C-7a), 137.1 (C-6), 133.3, 133.0, 128.9, 128.8, 114.9 (CN), 111.4 (C-4a), 87.5 (C-5), 86.6 (C-1′), 78.6 (C-4′), 74.3 (C-3′,), 73.2 (C-2′), 20.8 (OAc), 20.6 (OAc), 18.8 (C-5′); HRMS: calcd for C_23_H_22_N_5_O_6_ [M + H]^+^ 464.1565; found 464.1579.

### 4.7. Synthesis of 5′-deoxytoyocamycin (3) and 5′-deoxysangivamycin *(**4**)*

Ammonium hydroxide (45 mL) was added to a solution of 5 (1.80 g, 3.88 mmol) in 1,4-dioxane (18 mL). The reaction mixture was sealed and heated to 45 °C for 21 h. Then the reaction mixture was evaporated to dryness under reduced pressure and the residue was purified by silica gel column chromatography (CH_2_Cl_2_/MeOH 20:1, *v*:*v*) to afford compounds **3** (716 mg, 67%) and **4** (274 mg, 24%), respectively.

### 4.8. 5′-Deoxytoyocamycin *(**3**)*

R*_f_* = 0.2 (CH_2_Cl_2_/MeOH, *V/V* = 20:1); m.p. 190–192 °C (literature: 187–188 °C [16]); $\alpha_{D}^{\mathrm{25}}$ 87.88 (c = 0.099, DMSO); ^1^H-NMR δ 8.40 (s, 1H, H-8), 8.23 (s, 1H, H-2), 6.90 (s, 2H, NH_2_), 6.04 (d, *J* = 4.7 Hz, 1H, H-1′), 5.48 (d, *J* = 5.6 Hz, 1H, 2′-OH), 5.20 (d, *J* = 5.3 Hz, 1H, 3′-OH), 4.44–4.37 (m, 1H, H-2′), 4.00–3.91 (m, 1H, H-4′), 3.91–3.84 (m, 1H, H-3′), 1.30 (d, *J* = 6.3 Hz, 3H, CH_3_); ^13^C-NMR δ 157.5 (C-6), 154.1 (C-2), 150.8 (C-4), 132.9 (C-8), 115.8 (CN), 101.7 (C-5), 88.4 (C-1′), 83.8 (C-7), 80.2 (C-4′), 75.0 (C-3′), 74.2 (C-2′), 19.4 (CH_3_); HRMS: calcd for C_12_H_14_N_5_O_3_ [M + H]^+^ 276.1091; found 276.1099.

### 4.9. 5′-Deoxysangivamycin *(**4**)*

R*_f_* = 0.15 (CH_2_Cl_2_/MeOH, *V/V* = 10:1); m.p. 257–259 °C (literature value 260–262 °C [16]); $\alpha_{D}^{\mathrm{25}}$ −77.05 (c = 0.122, DMSO); ^1^H-NMR δ 8.09 (s, 2H, H-2, H-8), 7.99 (s, 1H, NH), 7.39 (s, 1H, NH), 6.08 (d, *J* = 4.2 Hz, 1H, H-1′), 5.50 (d, *J* = 5.5 Hz, 1H, 2′-OH), 5.20 (d, *J* = 5.7 Hz, 1H, 3′-OH), 4.24–4.19 (m, 1H, H-2′), 4.00–3.91 (m, 1H, H-4′), 3.91–3.84 (m, 1H, H-3′), 1.32 (d, *J* = 6.3 Hz, 3H, CH_3_); ^13^C-NMR δ 166.8 (C=O), 158.5 (C-6), 153.4 (C-2), 151.4 (C-4), 125.4 (C-8), 111.7 (C-7), 101.3 (C-5), 87.7 (C-1′), 79.5 (C-4′), 75.2 (C-3′), 74.8 (C-2′), 19.4 (CH_3_); HRMS: calcd for C_12_H_16_N_5_O_4_ [M + H]^+^ 294.1197; found 294.1208.

## Supplementary Materials

The NMR spectra of compound **3**–**4**, **6**, **9**, **11**–**12**, **14**, **16** are available online at http://www.mdpi.com/1420-3049/24/4/737/s1.
