# Supplementary material for: An Expeditious Total Synthesis of 5′-Deoxy-toyocamycin and 5′-Deoxysangivamycin
Source: Molecules. 2019 Feb 19;24(4):737. doi: 10.3390/molecules24040737 (PMC6413189; doi:10.3390/molecules24040737)
Supplement: Supplementary file 1 [file molecules-24-00737-s001.pdf]

Supporting Information

An Expeditious Total Synthesis of 5’-Deoxytoyocamycin and 5’-Deoxysangivamycin

Xiangyou Dong, Jie Tang, Chen Hu, Jiang Bai, Haixin Ding*, Qiang Xiao*

Key Laboratory of Organic Chemistry in Jiangxi Province, Institute of Organic Chemistry, Jiangxi Science & Technology Normal University, Nanchang 330013, China.Supporting Information

Supporting information for ^{1}H NMR and ^{13}C NMR Spectrum of compounds 5'-deoxytoyocamycin and 5'-deoxysangivamycin and intermediate compounds of **10**、**6**、**12**、**14**、**16**.

Contents

^{1}H NMR and 13C NMR Spectrum of compound 5' -deoxytoyocamycin .... S2、S3、S4

^{1}H NMR Spectrum of compound 5' -deoxysangivamycin S4

^{13}C NMR Spectrum of compound 5' -deoxysangivamycin S5

^{1}H NMR Spectrum of compound **10** S5

^{13}C NMR Spectrum of compound **10** S6

^{1}H NMR Spectrum of compound **6** S6

^{13}C NMR Spectrum of compound **6** S7

^{1}H NMR Spectrum of compound **12** S7

^{13}C NMR Spectrum of compound **12** S8

^{1}H NMR Spectrum of compound **14** S8

^{13}C NMR Spectrum of compound **14** S9

^{1}H NMR Spectrum of compound **16** S9

^{13}C NMR Spectrum of compound **16** S10

Table of Comparison NMR data for 5’-Deoxytoyocamycin between test value and literature value S11

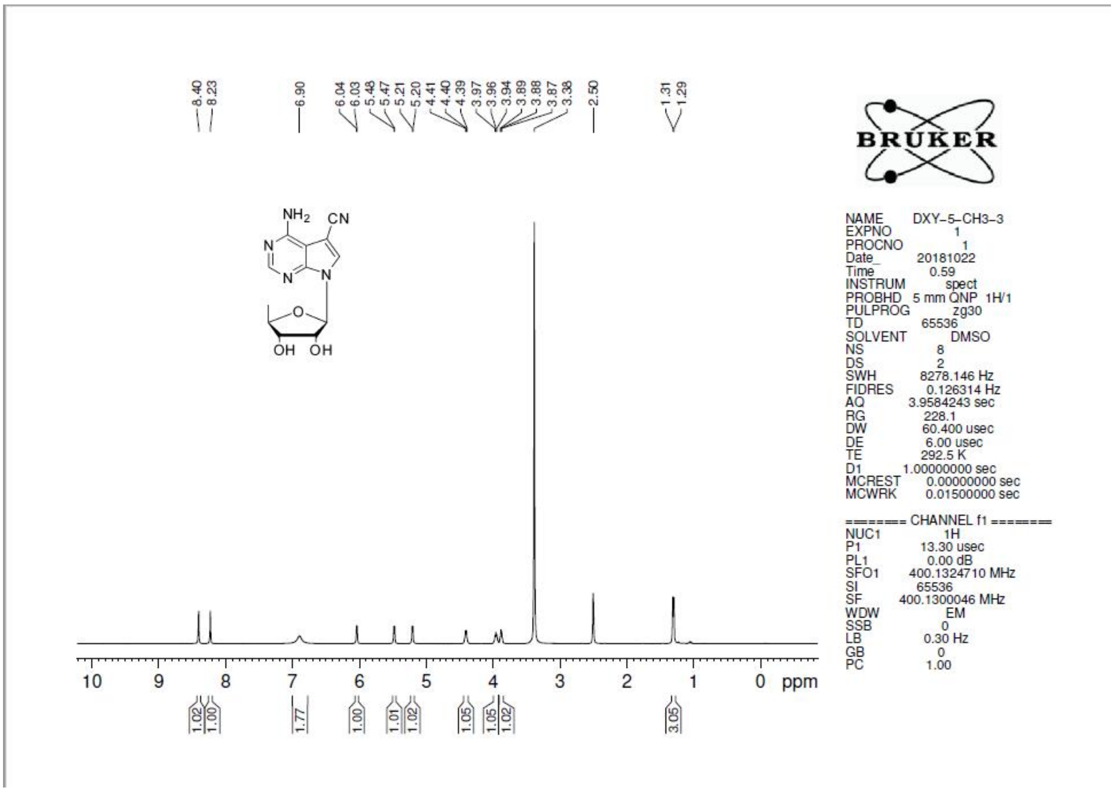

Figure S2 ^{1}H NMR Spectrum of compound 5' -deoxytoyocamycin

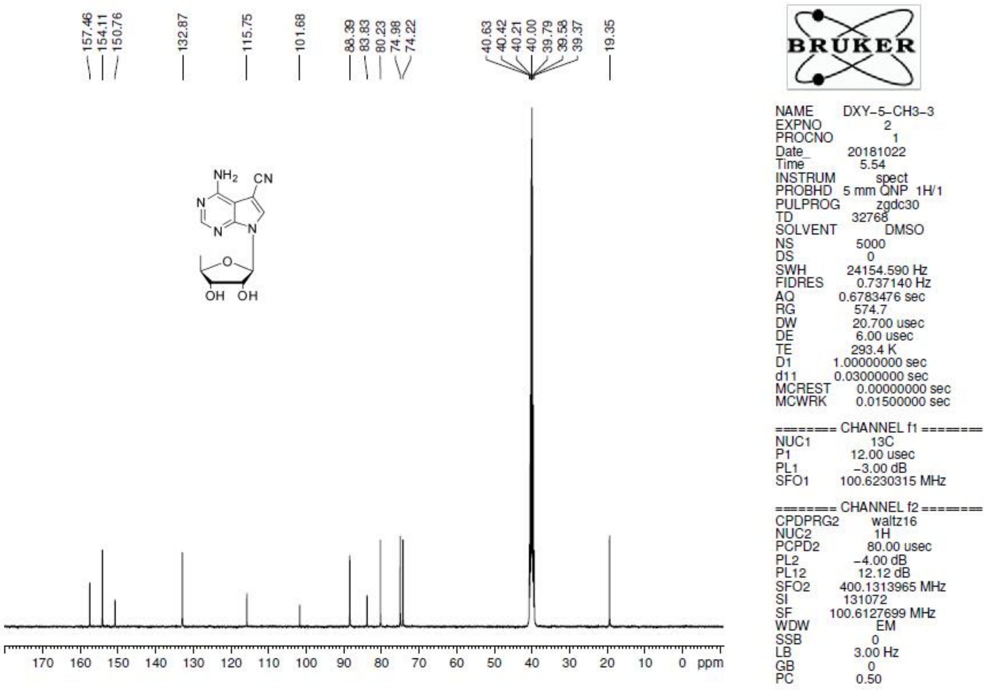

Figure S2 ^{13}C NMR Spectrum of compound 5' -deoxytoyocamycin

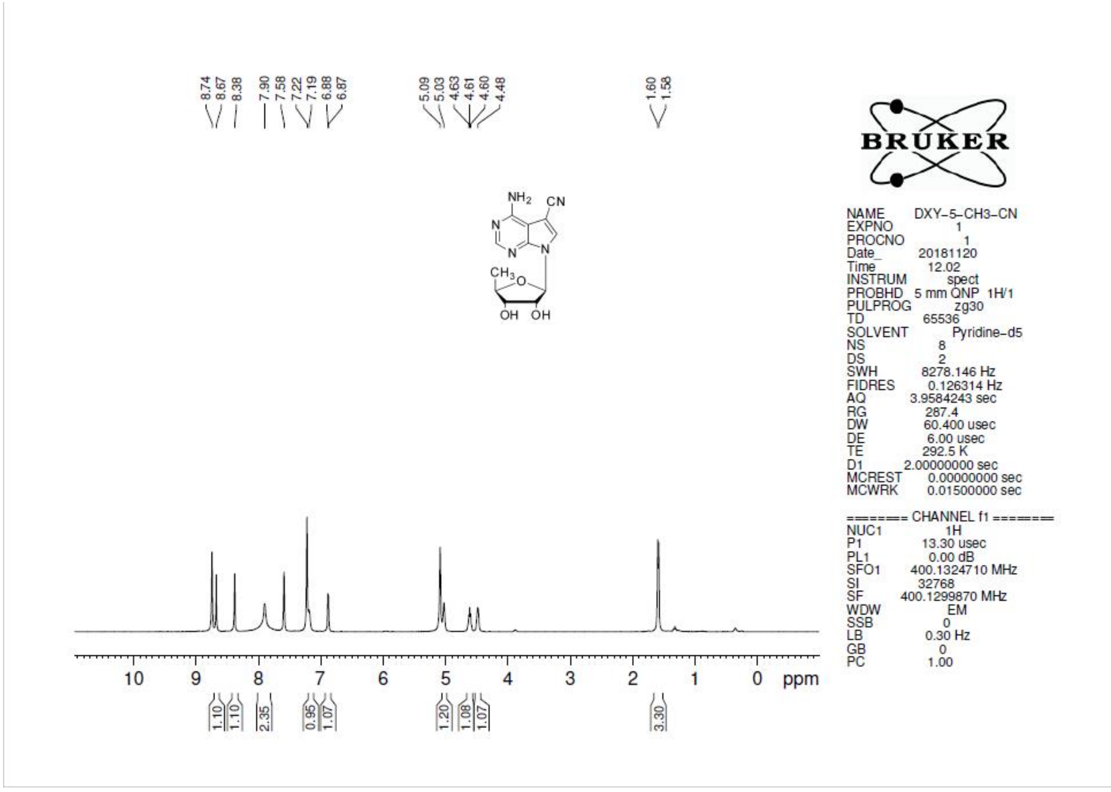

Figure S3 ^{1}H NMR Spectrum of compound 5' -deoxytoyocamycin

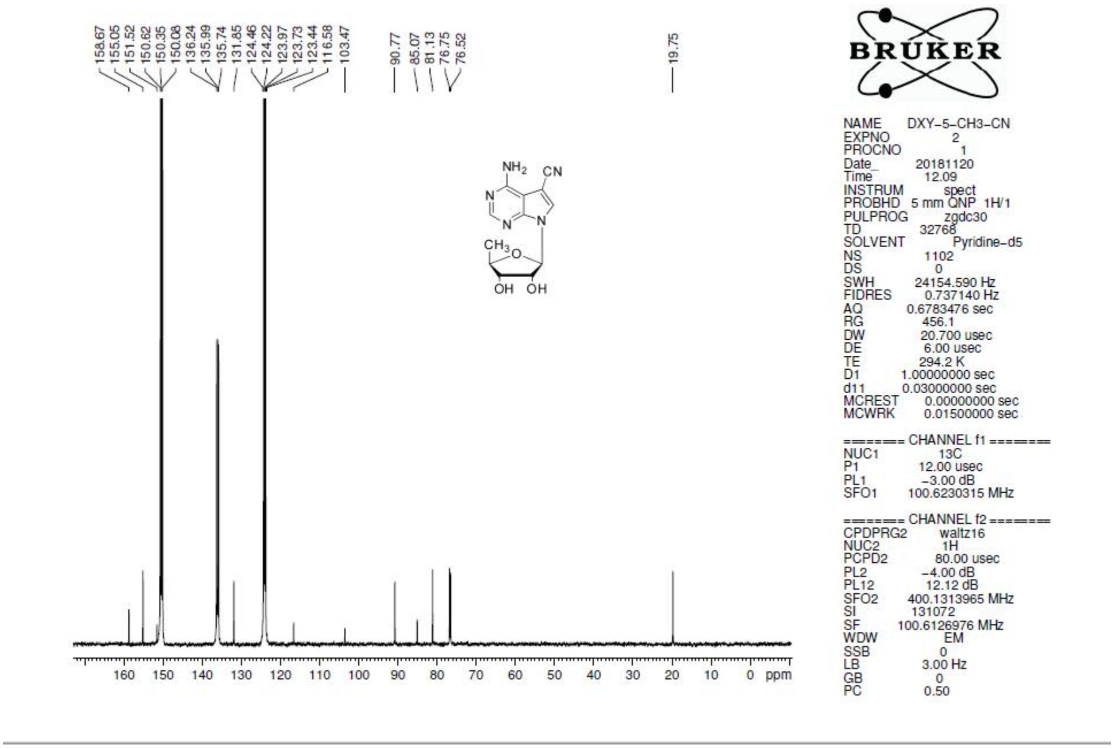

Figure S3 ^{13}C NMR Spectrum of compound 5' -deoxytoyocamycin

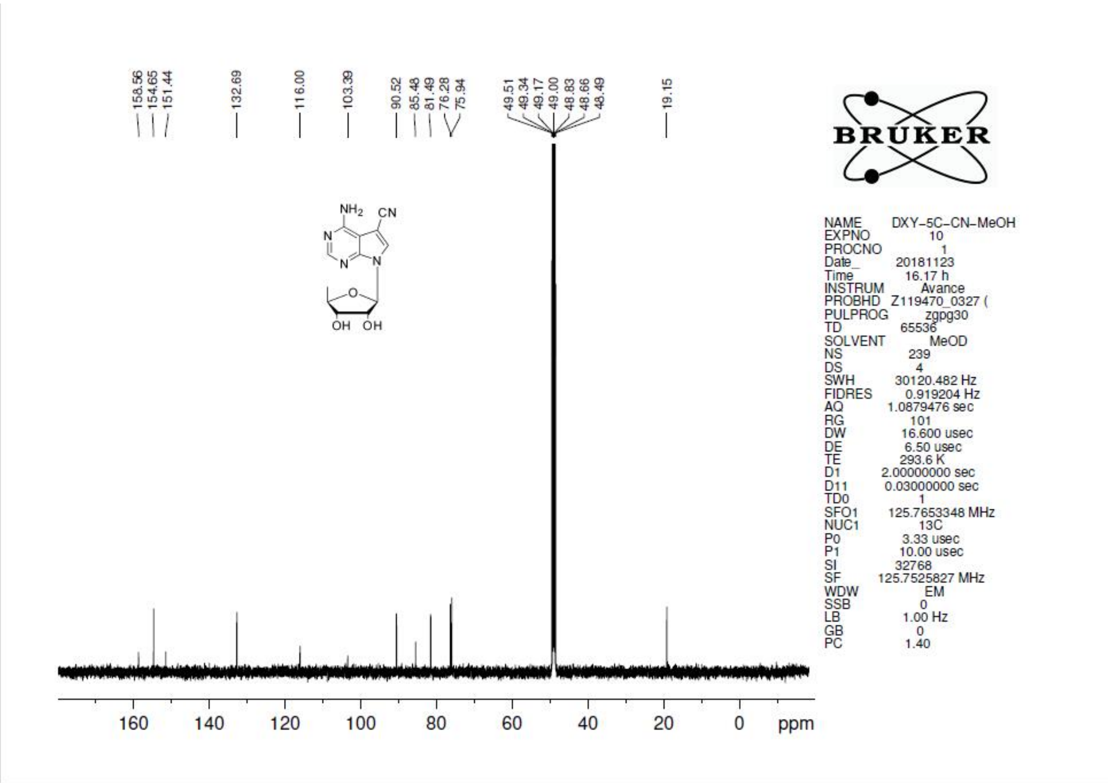

Figure S4 ^{13}C NMR Spectrum of compound 5'-deoxytoyocamycin

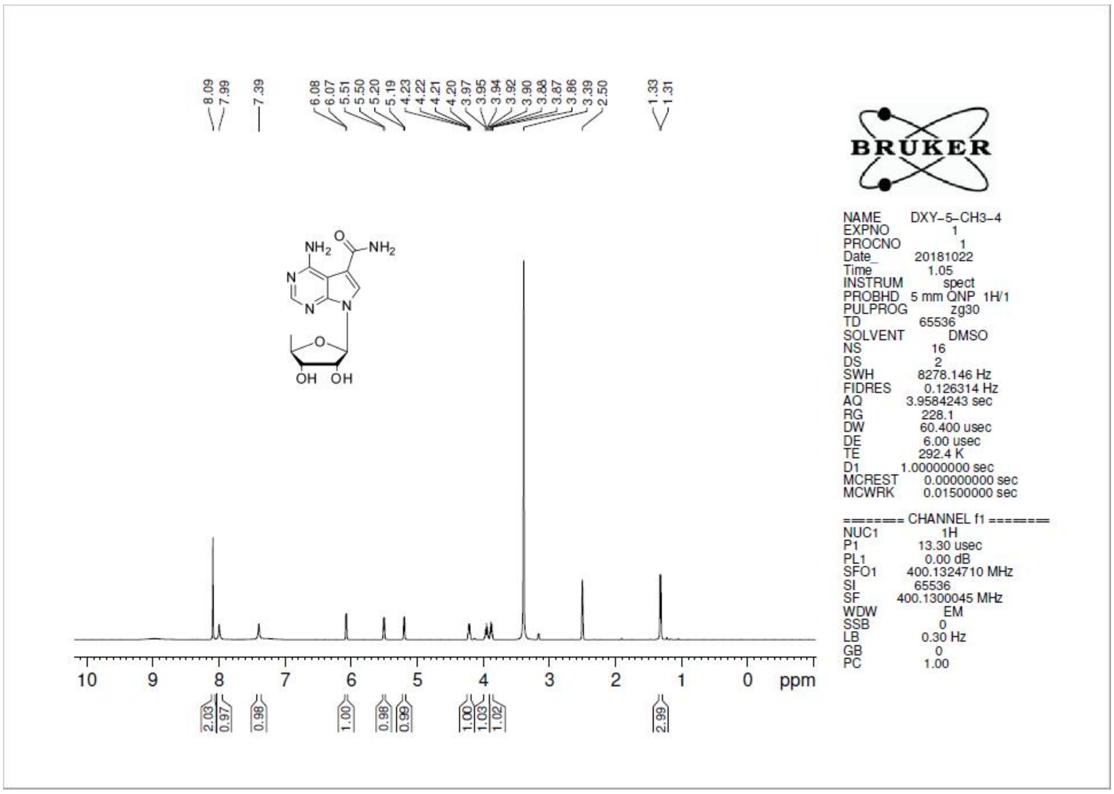

Figure S4 ^{1}H NMR Spectrum of compound 5' -deoxysangivamycin

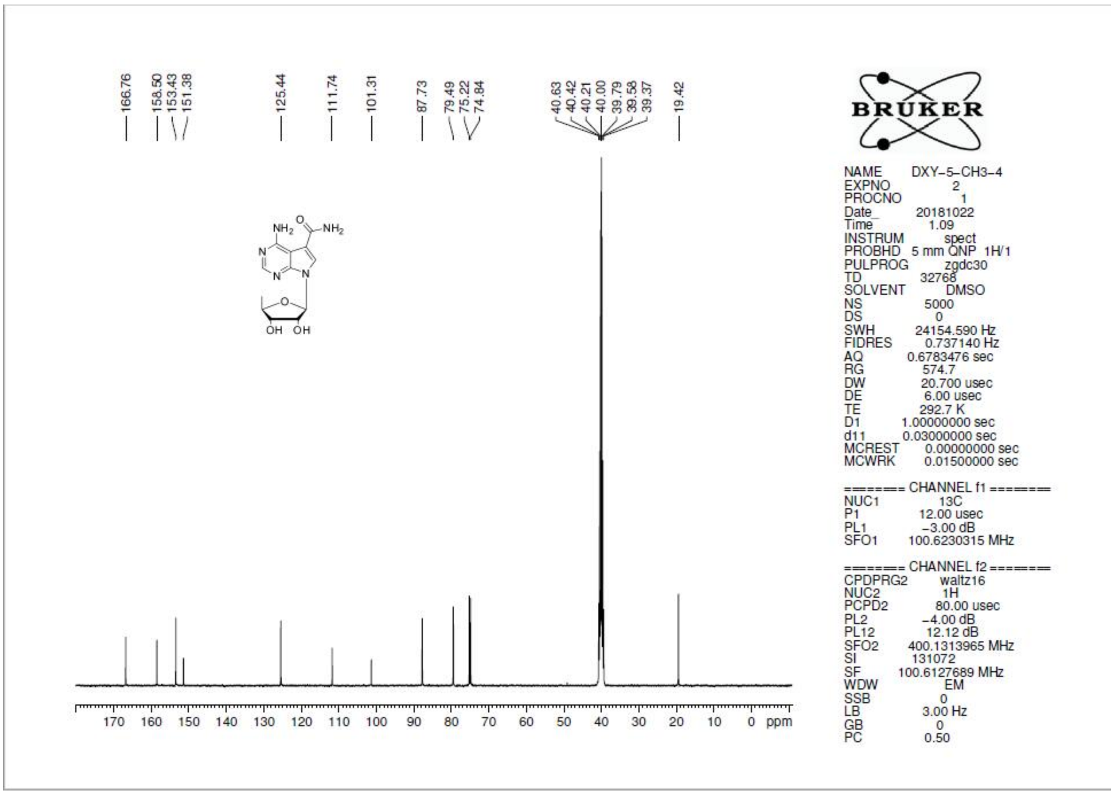

Figure S5 ^{13}C NMR Spectrum of compound 5' –deoxysangivamycin

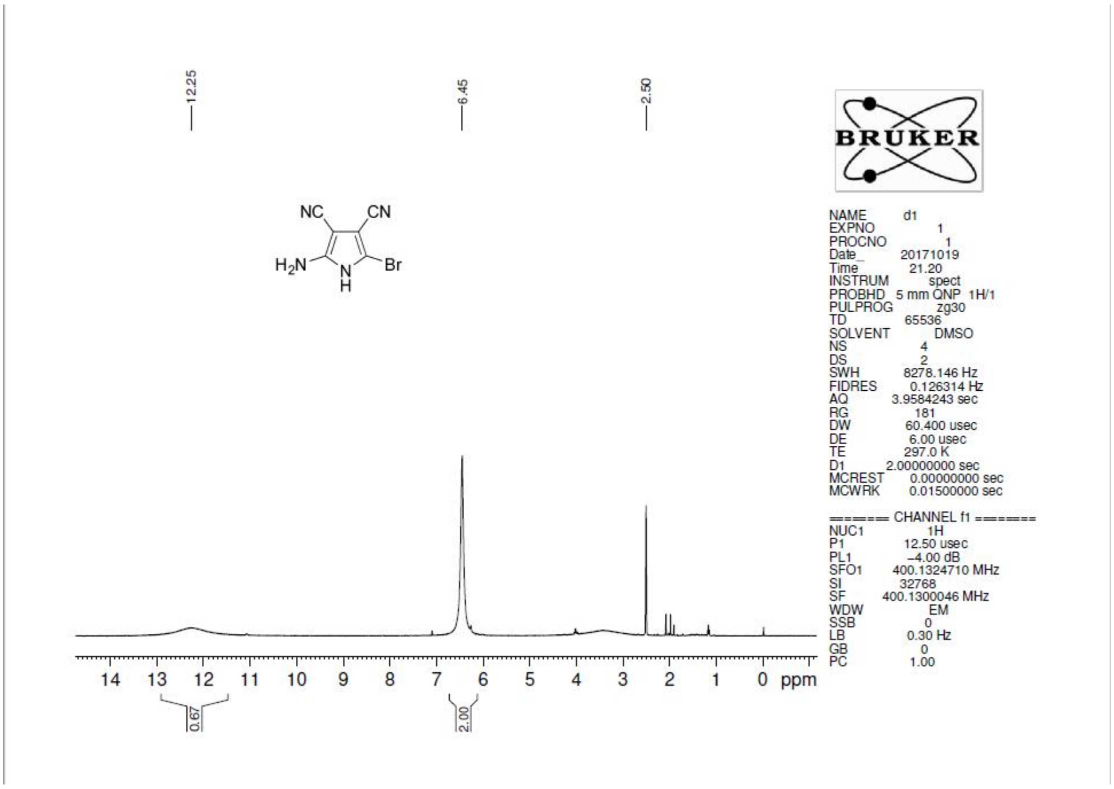

Figure S5 ^{1}H NMR Spectrum of compound **10**

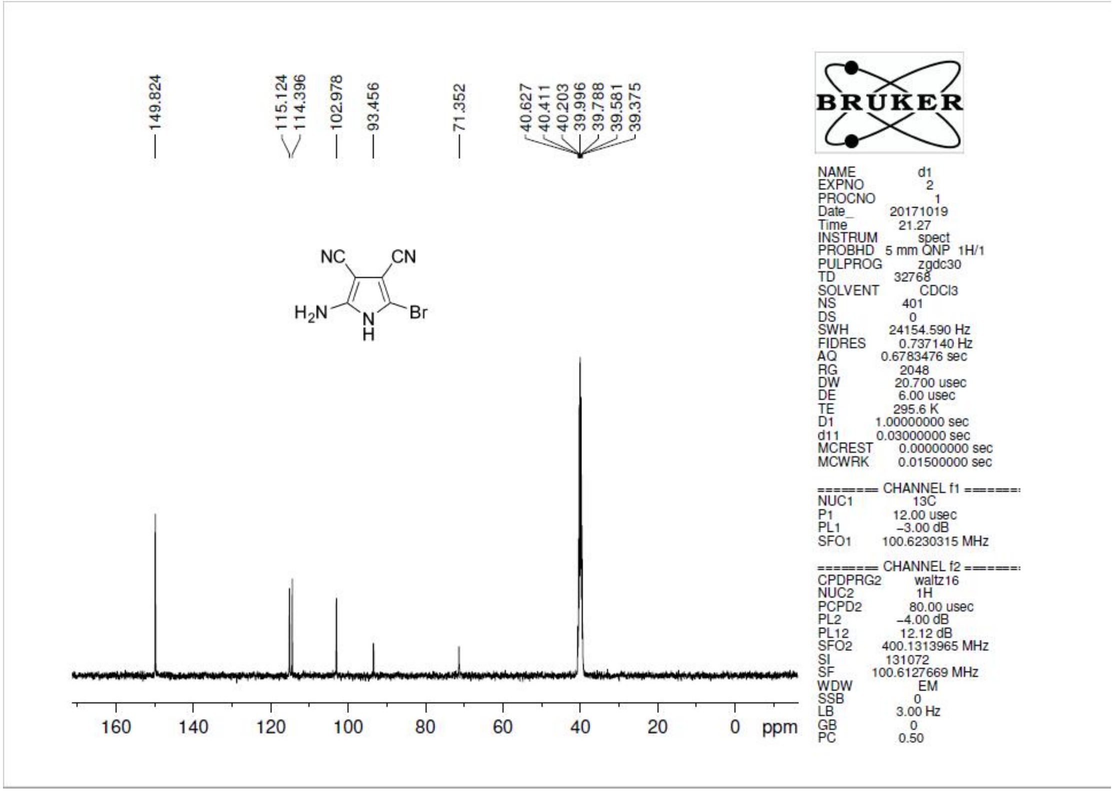

Figure S6 ^{13}C NMR Spectrum of compound **10**

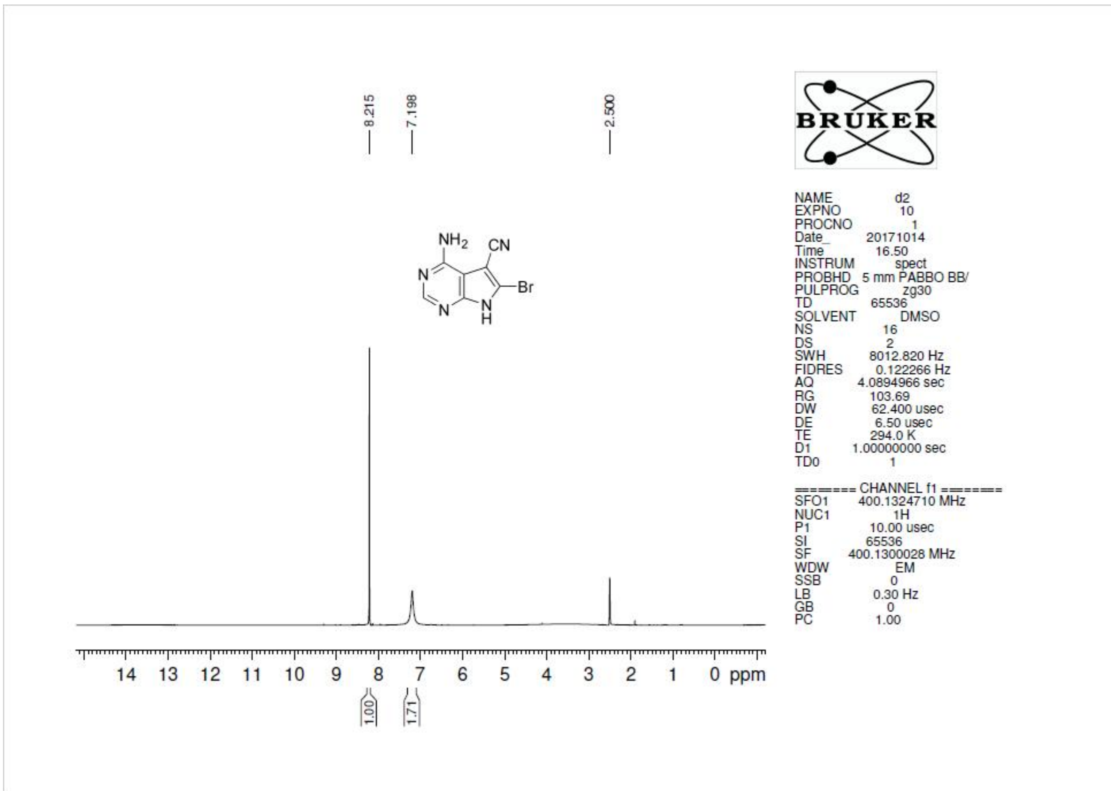

Figure S6 ^{1}H NMR Spectrum of compound **6**

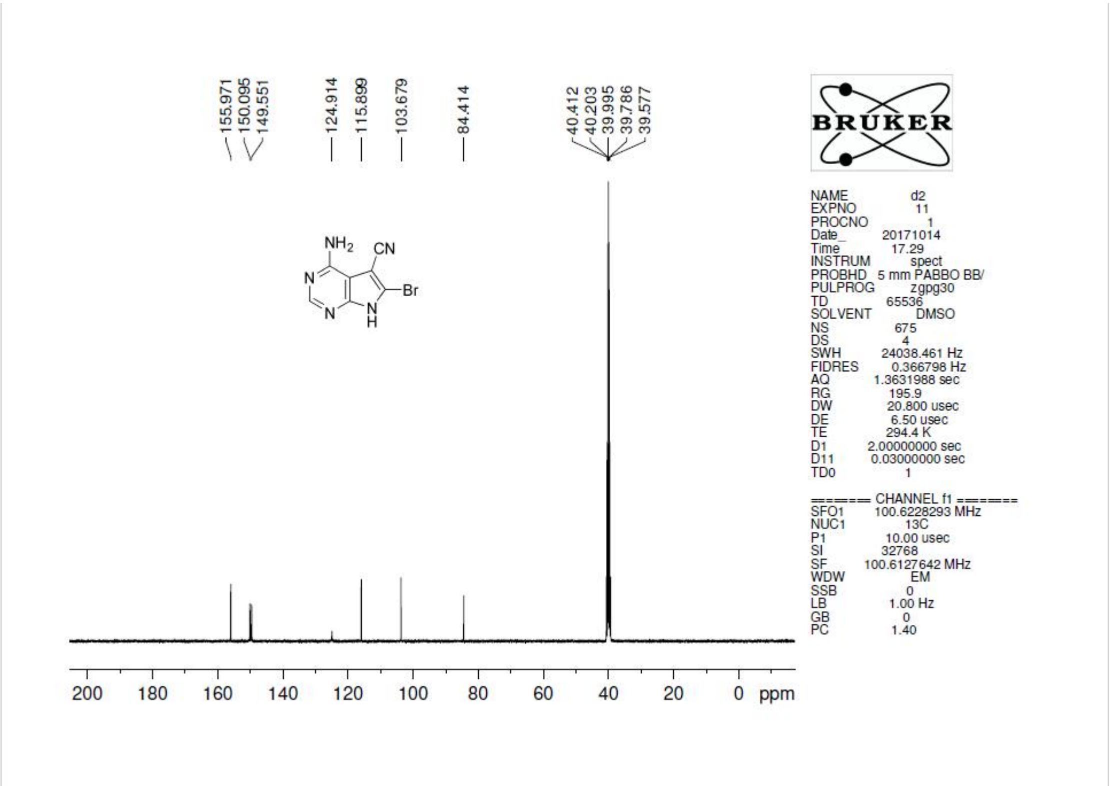

Figure S7 ^{13}C NMR Spectrum of compound **6**

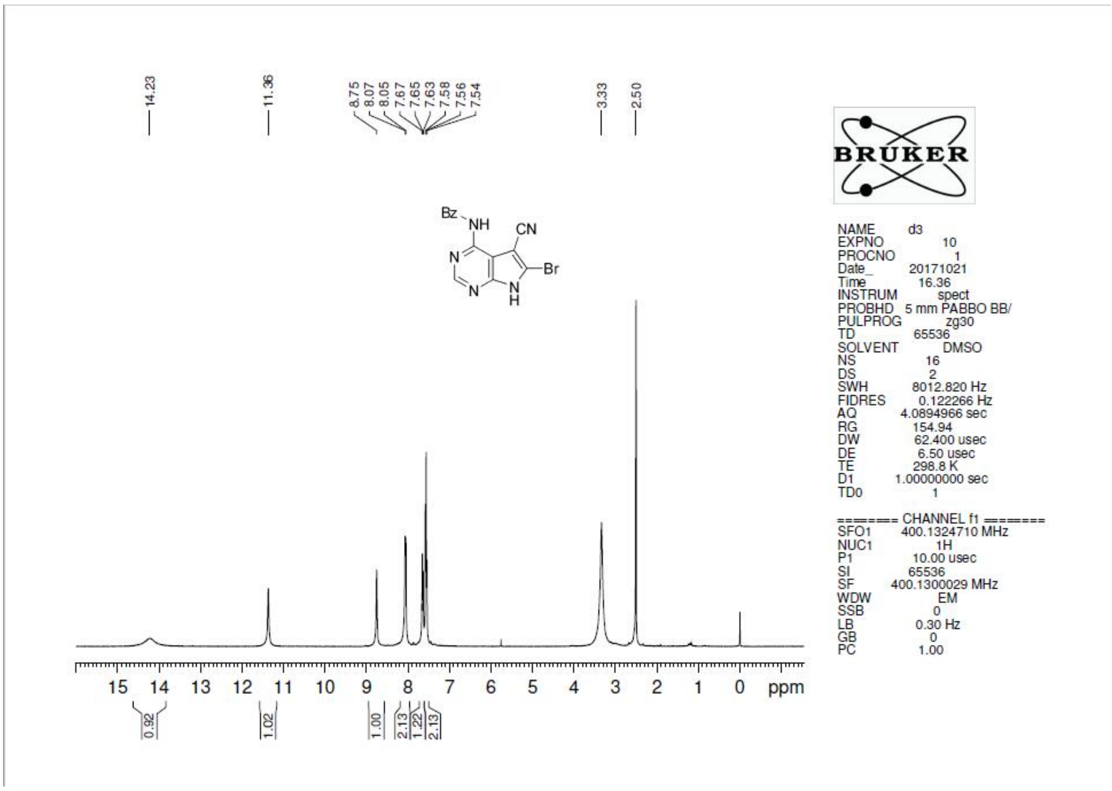

Figure S7 ^{1}H NMR Spectrum of compound **12**

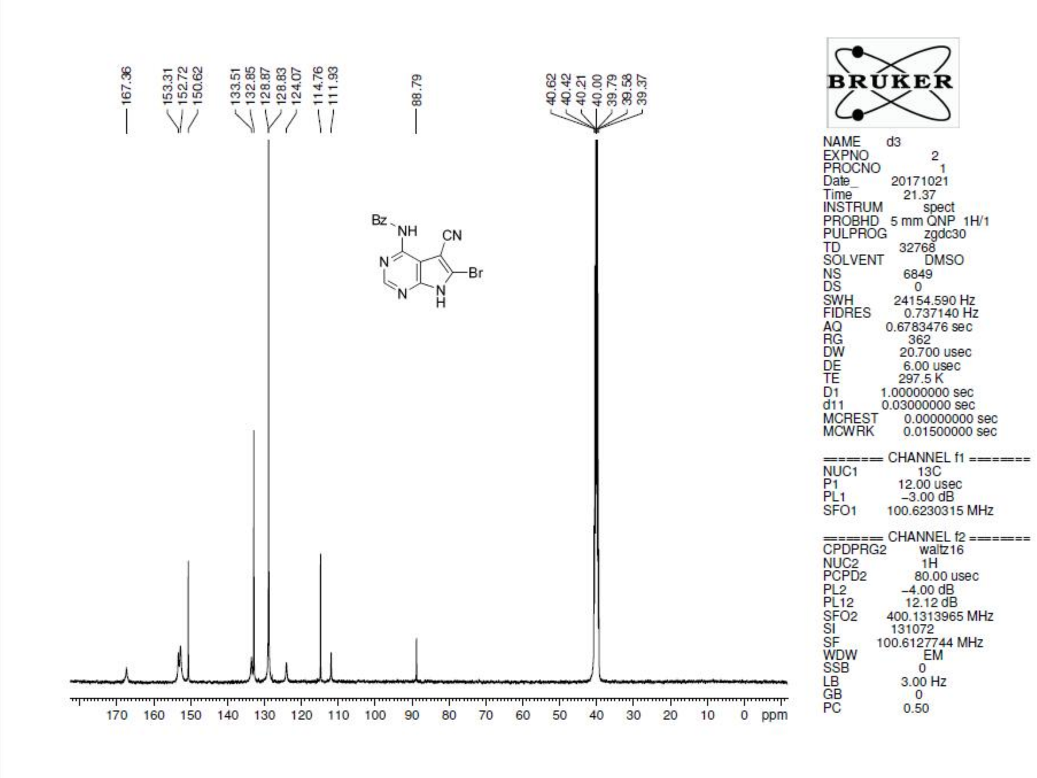

Figure S8 ^{13}C NMR Spectrum of compound **12**

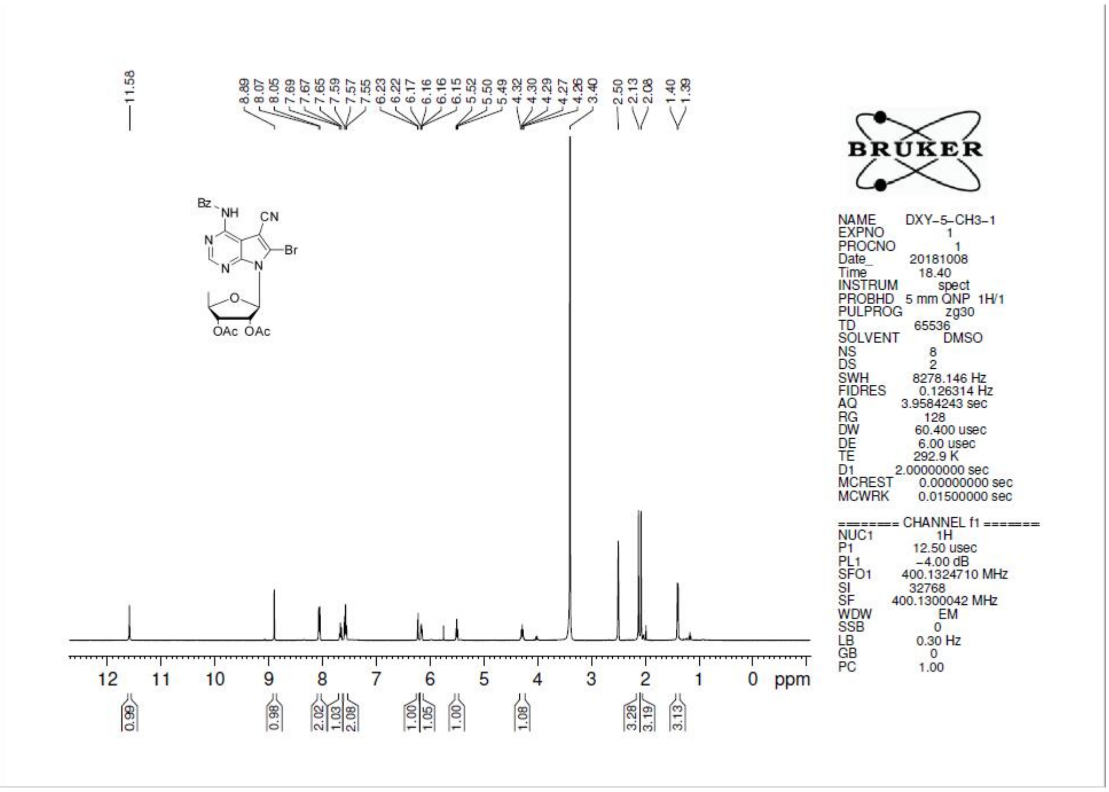

Figure S8 ^{1}H NMR Spectrum of compound **14**

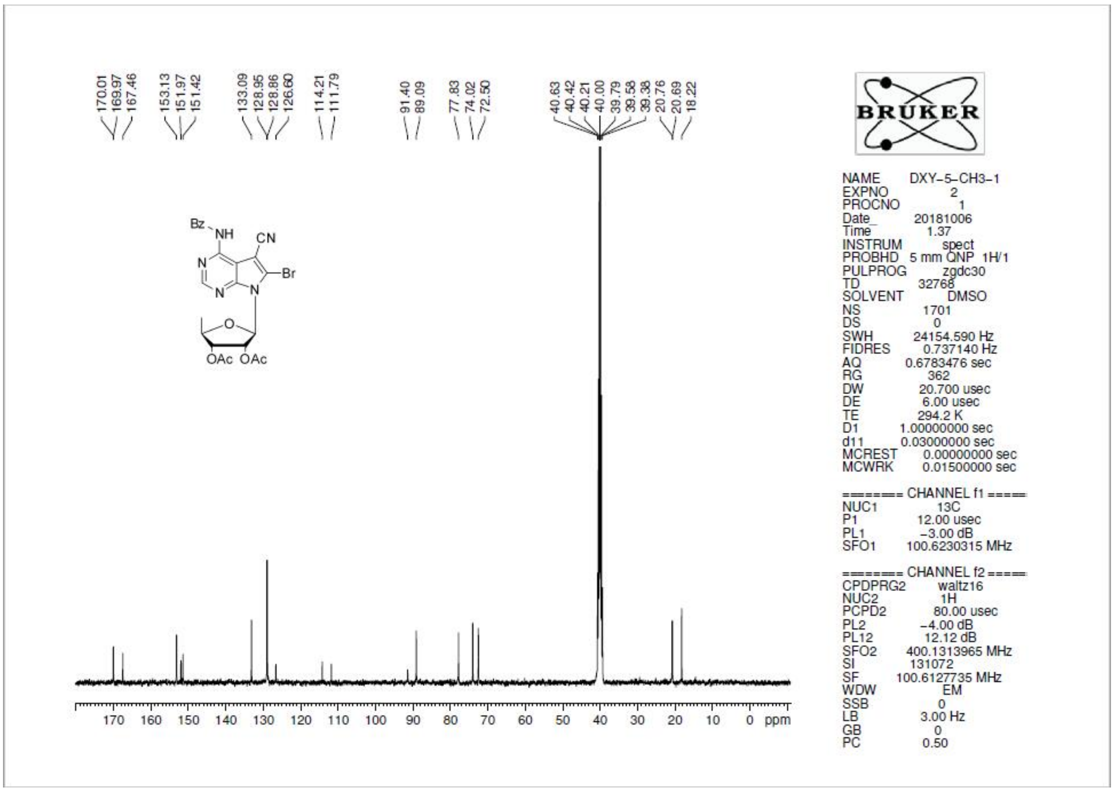

Figure S9 ^{13}C NMR Spectrum of compound **14**

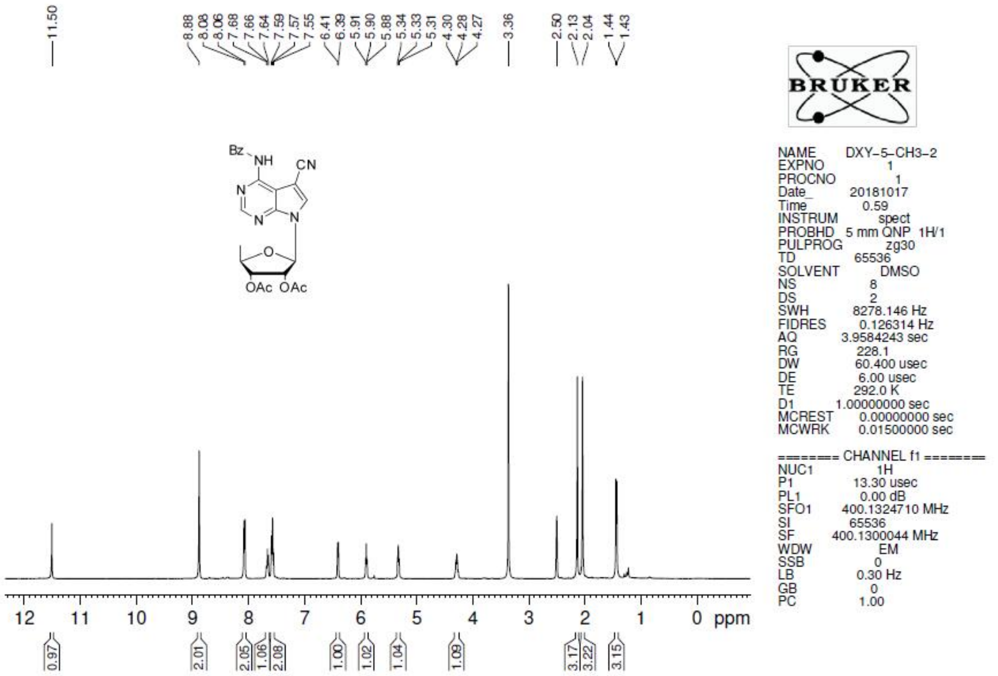

Figure S9 ^{1}H NMR Spectrum of compound **16**

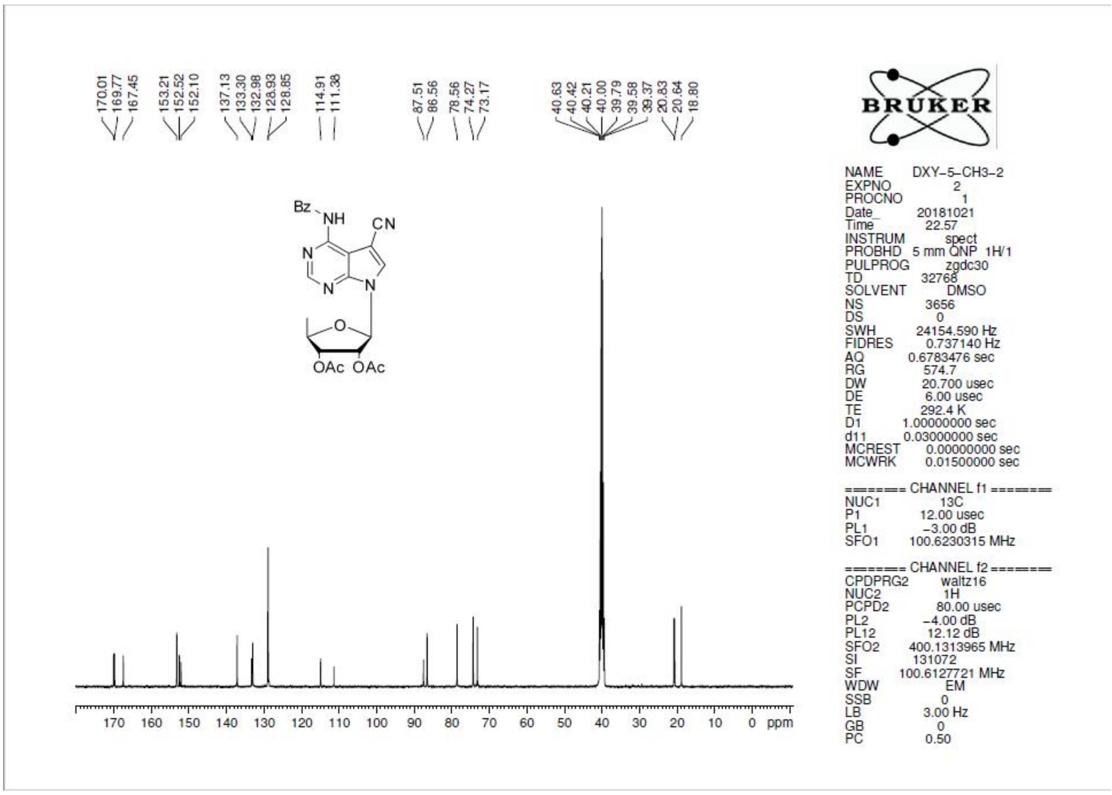

Figure S10 ^{13}C NMR Spectrum of compound **16**

Table 1. Comparison NMR data for 5' -Deoxytoyocamycin between test value and literature value (^{1}H NMR in pyridine-d_5, DMSO and ^{13}C NMR in CD_3OD,DMSO)

	literature value	test value	test value	literature value	test value	test value
position	^{13}C (CD_3OD)	^{13}C (CD_3OD)	^{13}C (DMSO)	^{1}H(J in Hz) (pyridine-d_5)	^{1}H(J in Hz) (pyridine-d_5)	^{1}H(J in Hz) (DMSO)
2	154.6	154.7 (CH)	154.1 (CH)	8.63 (s, 1H)	8.67 (s,1H)	8.23 (s, 1H)
4	151.5	151.4 (C)	150.8 (C)	-	-	-
5	-	103.4 (C)	101.7 (C)	-	-	-
6	158.6	158.6 (C)	157.5 (C)	-	-	-
7	85.5	85.5 (C)	83.8 (C)	-	-	-
8	132.7	132.7 (CH)	132.9 (C)	8.32 (s, 1H)	8.38 (s, 1H)	8.40 (s, 1H)
10	116.0	116.0 (CN)	115.8 (CN)	-	-	-
1′	90.6	90.5 (CH)	88.4 (CH)	6.83 (d, 3.9, 1H)	6.88 (d, 2.9, 1H)	6.04 (d, 4.7, 1H)
2′	75.9	76.0 (CH)	74.2 (CH)	4.98 (t, 4.8, 1H)	5.03 (s, 1H)	4.44–4.37 (m, 1H)
3′	76.3	76.3 (CH)	75.0 (CH)	4.44 (t, 5.4, 1H)	4.48 (s, 1H)	3.91–3.84 (m, 1H)
4′	81.5	81.5 (CH)	80.2 (CH)	4.57 (dq, 6, 6.3, 1H)	4.66–4.56 (m, 1H)	4.00–3.91 (m, 1H)
5′	19.1	19.2 (CH_3)	19.4 (CH_3)	1.59 (d, 6.6, 1H)	1.59 (d, 6.0, 3H)	1.30 (d, 6.3, 3H, CH_3)
NH_2				7.81 (br, s, 2H)	7.90 (br, s, 2H)	6.90 (br, s, 2H)
OH				-	7.19 (s, 1H)	5.48 (d, 5.6, 1H) 5.20 (d, 5.3, 1H)
